# Integrative Priming of Compositional and Locative Relations

**DOI:** 10.3389/fpsyg.2017.00359

**Published:** 2017-03-15

**Authors:** Lara L. Jones, Lee H. Wurm, Ryan D. Calcaterra, Noa Ofen

**Affiliations:** ^1^Department of Psychology, Wayne State UniversityDetroit, MI, USA; ^2^Institute of Gerontology, Wayne State UniversityDetroit, MI, USA

**Keywords:** integrative priming, Embodied Conceptual Combination, Complementary Role Activation, thematic relations, compositional relations, locative relations

## Abstract

Integrative priming refers to the facilitated recognition of a target word (*bench*) as a real word following a prime (*park*). Prior integrative priming studies have used a wide variety of integrative relations including temporal (*summer rain*), topical (*travel book*), locative (*forest river*), and compositional (*peach pie*) relations. Yet differences in the types of integrative relations may yield differences in the underlying explanatory processes of integrative priming. In this study, we compared the magnitude, time course, and three theoretically based correlates of integrative priming for compositional (*stone table*) and locative (*patio table*) pairs in a lexical decision task across four stimulus onset asynchronies (SOAs; 50, 300, 800, and 1,600 ms). Based on the Complementary Role Activation theory, integrative ratings (the extent to which the prime and target can be combined into a meaningful phrase) were predicted to facilitate target RTs. Based on the Embodied Conceptual Combination (ECCo) theory, the local co-occurrence of the prime and target, and the ability to perceptually simulate (visually experience) the prime-target pair were tested as predictors. In comparison to unrelated pairs (*nose table*), target RTs were faster for the compositional and locative pairs, though did not differ between these relations. In support of the Complementary Role Activation theory, integrative ratings predicted target RTs above and beyond our control variables. In support of the ECCo theory, co-occurrence emerged as an early predictor of target RTs, and visual experience ratings was a reliable predictor at the 300 ms SOA, though only for the compositional relations.

## Introduction

Relational integration refers to the process by which two nouns can be combined via the inference of a sensible relation into a distinct and plausible entity that denotes a subclass of the second noun. For example, *island house* is rapidly interpreted as a house that is *located* on an island and thus denotes a specific type of house. Such relational integration facilitates online word recognition in a lexical decision task (henceforth “integrative priming”; Estes and Jones, 2009; Jones and Golonka, 2012) and in masked perceptual identification (Mather et al., 2014) as well as facilitating memory in recognition (Jones et al., 2008) and in cued recall (Badham et al., 2012). Prior relational integration research has included a mixture of integrative relations including locative (*mountain–snow*), compositional (*wool–coat*), temporal (*winter–sport*), topical (*travel–book*), and whole/part (*monkey–foot*). Yet several researchers (McRae and Boisvert, 1998; Hutchison, 2003; Jones and Estes, 2012) have noted the importance of moving beyond broad categories of semantic relations (taxonomic, thematic, integrative) to investigate more specific relations. Indeed, some studies have focused on the accessibility of a specific and single type of relation within a given experiment (*compositional*: Estes and Jones, 2006; *causal*: Fenker et al., 2005; *locative, instrumental, event*: Hare et al., 2009). Just as broader relational categories contribute to a word's semantic richness (i.e., variability in the information associated with a word's meaning; Yap et al., 2011), specific relations also facilitate a word's accessibility. For instance, the greater the number of locations generated for a concrete noun, the faster the lexical decision latencies were for that noun (Recchia and Jones, 2012). Accordingly, our purpose was to compare and contrast the time course, magnitude, and underlying predictors of lexical priming for integrative pairs requiring the inference of a compositional (*log*–*house*) vs. a locative (*island*–*house*) relation. We chose to compare these two relations in part due to their ubiquity in studies of conceptual combination, integrative priming, and thematic relations (e.g., Estes and Jones, 2006, 2009; Hare et al., 2009; Estes et al., 2011; Jones and Golonka, 2012; Jouravlev and McRae, 2016). As discussed further, our chosen underlying predictors of integrative priming stem from two likely explanatory theories, namely, Complementary Role Activation, (Mather et al., 2014) and Embodied Conceptual Combination (ECCo, Lynott and Connell, 2010).

Target words (*curtain*) are recognized faster in a lexical decision task (LDT) following a prime word with which it can be combined (*velvet–curtain; theater–curtain*) than following a neutral symbol (^*****^–*curtain*; Estes and Jones, 2009) or an unrelated word (*hammer–curtain*). Such integrative priming has been found independent of several other factors known to facilitate priming including: association strength (i.e., the proportion of a large participant sample producing the target word in response to the prime as a cue in a free association task), feature similarity, the familiarity of the prime target combination, or the co-occurrence in written language (Estes and Jones, 2009; Jones and Golonka, 2012; Mather et al., 2014). The magnitude and prevalence of integrative priming is similar to that found for semantic (taxonomic) priming between featurally similar primes and targets (Estes and Jones, 2009; Badham et al., 2012; Jones and Golonka, 2012). Moreover, reliable integrative priming has been found in LDTs across a wide range of stimulus onset asynchronies (SOAs; i.e., delay between prime and target onsets) from 100 to 2,500 ms (Estes and Jones, 2009; Jones and Golonka, 2012). In addition to these results using LDTs, results of a masked perceptual identification task and a Stroop color naming task further demonstrated the robust and uncontrollable nature of integrative priming (Mather et al., 2014). From these integrative priming results in conjunction with studies showing relation priming between conceptual combinations sharing the same relation (e.g., faster comprehension of *straw hat* following *steel scissors*; Estes and Jones, 2006), the Complementary Role Activation theory was proposed to explain integrative priming. Specifically, prime and target words automatically activate their respective and complementary relational roles (e.g., material for *velvet*; object for *curtain*). In turn, these relational roles instantiate a specific relation (e.g., composition) that serves to combine the prime and target into a subtype of the target (e.g., a *velvet curtain* is type of *curtain* that is composed of *velvet*). In contrast, for an unrelated pair (e.g., *hammer curtain*), there would be no complementary relational roles that would be activated for *hammer* and *curtain*. The target then must be evaluated to determine whether it indeed meets the constraints of the role activated by the prime (e.g., Is it plausible for a curtain to be composed of velvet?). This final plausibility check would occur retrospectively (following target presentation), whereas the activation of the prime's relational role (material) and the relation (composed of) would occur prospectively (prior to target presentation). If the relational roles activated by either concept are not complementary, as is likely the case for the unrelated pair *hammer curtain*, then there would be no reason to check the plausibility of the prime-target pair. Following from this theory, the offline judgment of the extent to which two concepts can be linked together to produce a sensible phrase (i.e., the integrative rating) should predict integrative priming (i.e., faster target latencies following integrative than unrelated primes).

In addition to integrative ratings, the ability to simulate or form a plausible image of the prime-target combination including the locative or compositional relation for that combination may also contribute to integrative priming effects. As described by Barsalou's perceptual symbol systems theory (Barsalou, 1999), when people represent concepts, such as a *house*, they represent it with multi-modal simulations of their experiences of a *house* and include background information as part of the simulation (e.g., the neighborhood or other scenery surrounding the house; see McRae and Jones, 2013). Perceptual simulation would be especially involved for strongly perceptual (i.e., more concrete) concepts like *chair* or *house* in comparison to weakly perceptual ones like *republic* or *factor* (Barsalou and Wiemer-Hastings, 2005; Connell and Lynott, 2012). Indeed, a relatively shallow lexical decision task (LDT) can serve to facilitate situational representations of perceptually strong concepts (Connell and Lynott, 2012). Conceptual combinations (*log house, island house*) entail simulations of individual concepts that are combined to form more complex simulations (Wu and Barsalou, 2009). Whereas, isolated concepts would entail a broad representation of situational information, the modifier in conceptual combinations would serve to focus the simulation on the basis of that modifier. Barsalou (1999, 2003) proposes that the relations, such as compositional and locative ones connecting the modifying noun with the head noun are implemented via relational simulators (e.g., the house is made of logs; the house is located on an island). In turn, the features that could be listed for the combination reflect this simulation. For example, Wu and Barsalou found more internal features (dirt and roots) listed for the noun phrase *rolled-up lawn*, in comparison to just the head noun *lawn*, for which the properties were more external (green, blades) and situational (you play on it).

The Embodied Conceptual Combination theory (ECCo; Lynott and Connell, 2010) proposes a perceptual simulation process for conceptual combinations as well as a “quick and dirty” linguistic system for more lexicalized combinations (Connell and Lynott, 2011). The linguistic system relies on the speedy lexical retrieval of previously encountered word pairs, whereas the perceptual system may require a slow generation of a novel combination (*octopus apartment*) or the retrieval of a recently encountered image reflecting a lexicalized pair. For example, though a more lexicalized (as opposed to novel) combination like an *office chair* may rely on both linguistic and perceptual simulation information, the frequent co-occurrence of *office* and *chair* as a combination would make it more easily and more quickly retrievable and less reliant on the slower simulation processes. The extent to which linguistic vs. perceptual factors play a role in the interpretation or relational integration of two concepts depends on both the nature of the task (e.g., spatial judgments vs. linguistic judgments) and the stimuli (e.g., words vs. pictures), with a greater influence for linguistic factors like co-occurrence in more verbal tasks, such as an LDT (Louwerse and Jeuniaux, 2010). Across all types of tasks though, both linguistic representations and perceptual simulation measures should predict performance in an LDT, with linguistic representations (co-occurrence) emerging earlier as a predictor at shorter SOAs (Louwerse and Jeuniaux, 2010; Louwerse and Connell, 2011; Louwerse and Hutchinson, 2012). The Language and Situated Simulation (LASS) theory also proposes that linguistic information may precede simulation, but in terms of the peak of activation for that process rather than the initial emergence of it (Barsalou et al., 2008). So then the linguistic co-occurrence of *velvet* with *curtain* would facilitate processing prior to the facilitation produced by forming an image of a *velvet curtain*. For unrelated pairs (*hammer curtain*), the linguistic shortcut (i.e., lack of co-occurrence) leads to faster rejection of the combination as being sensible (Connell and Lynott, 2013). In a LDT, this may result in lower accuracies for the lexical decisions on the unrelated primed targets.

In sum, given prior lexical priming for integrative pairs across a wide range of SOAs from 100 to 2,500 ms (Estes and Jones, 2009; Jones and Golonka, 2012), we predicted reliable lexical priming at each SOA with faster RTs for the targets following the compositional and locative primes than following the unrelated primes. Both of these integrative pair types are quite common, and we had no basis for predicting a difference between them in the magnitude or time course of priming. However, we conducted additional analyses to determine the extent to which co-occurrence, perceptual (visual) experience, and integrative ratings differentially predicted priming for locative vs. compositional pairs across four SOAs. Our inclusion of four widely ranging SOAs (50, 300, 800, and 1,600 ms) served three purposes. First, it enabled us to replicate Lourwerse and colleagues' prior findings of an earlier influence for linguistic than perceptual factors. Second, we sought to extend prior integrative priming studies by investigating the emergence of priming at an even earlier SOA (50 ms) as opposed to 100 ms SOA. Third, with the inclusion of the longer SOAs, we were able to better assess the point at which the influence of co-occurrence, perceptual simulation, and relational integration diminished.

## Methods

### Participants

Wayne State University undergraduates (*N* = 355, *M*_*age*_ = 22.14, *SD*_*age*_ = 6.60, females 53.8%) participated in the study for partial course credit, and were divided among the 50 ms (*n* = 106), 300 ms (*n* = 77), 800 ms (*n* = 92), and 1,600 ms (*n* = 80) SOA conditions.

### Stimuli

For each of 48 real word targets, we created locative, compositional, and unrelated primes (see Appendix). We retrieved from the English Lexicon Project (Balota et al., 2007, http://elexicon.wustl.edu/) several variables for the primes and targets that are known to influence word recognition in a lexical decision task including: length, the baseline LDT RT (ELP RT); the standardized baseline LDT prime RT (ELP zRT), and logarithmic contextual diversity (Adelman et al., 2006; Brysbaert and New, 2009). We also retrieved age-of-acquisition (AoA) ratings for both prime and target (Kuperman et al., 2012).

For the prime-target pairs, we assessed three predictors: co-occurrence, integratability, and the extent to which the pair could be perceptually experienced in the visual modality (i.e., visual experience ratings). In addition to these pair variables, forward association strengths (FAS) and backward associative strengths (BAS) also influence the magnitude of lexical priming (Lucas, 2000; Hutchison, 2003; Hutchison et al., 2008; Jones, 2012). So we obtained association strengths from the University of South Florida Free Association norms (Nelson et al., 1998, 2004). Because both forward and backward association strengths are robust predictors of lexical priming, most of our primes were intentionally chosen to be weakly associated so that the influence of association strength would not overshadow that of our predictors. Association strengths were highly skewed for our related pairs (Locative Skewness: FAS = 4.08, BAS = 4.84; Compositional Skewness: FAS = 3.16, BAS = 3.40), with approximately 85% of the items within each related pair type as no more than weakly associated (association strengths <0.10).

Co-occurrence between primes and targets also influence lexical priming, particularly at shorter SOAs (≤ 200 ms; e.g., Jones, 2012; Jones and Golonka, 2012). To assess the co-occurrence of the prime-target pair, we used a method similar to that used in other conceptual processing studies (Louwerse and Connell, 2011; Connell and Lynott, 2013). The frequency distribution of each word pair was calculated using word counts from the Web 1T 5-gram database (Brants and Franz, 2006). The Web 1T database is a massive corpora that consists of over a trillion tokens collected by Google Research. We used the database to measure the bigram, trigram, 4-gram, and 5-gram frequency of each word pair, and to calculate bidirectional 5-gram frequencies of co-occurrence between prime and target for each nominal pair. The algorithm for calculating bidirectional frequency is the natural log (*ln*) transformation of the sum of the hits for the word pair forward and backward, plus the number of hits when one, two, or three words intervened. This measure of bidirectional co-occurrence was advantageous in comparison to our previously used Google hits measure of “local co-occurrence” (Jones and Golonka, 2012; Mather et al., 2014). Specifically, the bidirectional N-gram co-occurrence measure with up to three intervening words between prime and target was less restrictive than using Google hits, which measured the co-occurrence of only the forward ordered pair with no intervening words.

Separate groups of undergraduates at Wayne State University rated the integratability (*N* = 24) of all 144 prime–target pairs and the perceptual experience (*N* = 35) of the 96 related (compositional and locative) pairs. Integratability was rated as the extent to which each prime–target pair could be linked together to form a sensible phrase on a scale from 1 (not linked) to 7 (tightly linked; cf. Estes and Jones, 2009). Perceptual experience ratings were used as a measure of perceptual simulation. We focused on the visual modality and adapted the instructions used for individual concepts (Lynott and Connell, 2009, 2013; Connell and Lynott, 2012) to apply to conceptual combinations. Our instructions stated:

In this study we are interested in how people experience everyday objects only by sight as opposed to also experiencing them using other senses (hearing, smelling, tasting, touching). For each of 96 items, please rate the extent to which you fully experience that item using only your visual sense (only by seeing) using the following scale from 0 (not at all) to 5 (greatly). Using the numberpad (right side of the keyboard), indicate your response by entering a 0, 1, 2, 3, 4, or 5. There are no right or wrong answers, just use your best judgment. You may find that some items are easier to experience visually than are other items, so PLEASE USE THE FULL RANGE OF THE SCALE IN MAKING YOUR RESPONSES.

Descriptive statistics for these pair variables and prime characteristics are shown by Prime-type in Table 1 along with a repeated-measures ANOVA assessing differences on each variable across the Prime-types.

**Table 1:** **Descriptive statistics for prime characteristics and pair variables**.

	**Compositional**	**Locative**	**Unrelated**	**ANOVA**	**Comparison across prime-types**
**PRIME CHARACTERISTICS**					
Length	5.46 (1.68)	5.73 (1.77)	5.31 (1.26)	*F* = 0.97, *p* = 0.38	Comp = Loc = Unrel
ELP RT	631 (70)	617 (45)	599 (49)	*F* = 3.81, *p* < 0.05	(Comp = Loc) > Unrel
ELP zRT	−0.55 (0.21)	−0.59 (0.15)	−0.67 (0.15)	*F* = 6.60, *p* < 0.01	(Comp = Loc) > Unrel
CD	2.49 (0.52)	2.87 (0.42)	2.87 (0.51)	*F* = 9.03, *p* < 0.001	Comp > (Loc = Unrel)
Ortho N	4.96 (4.72)	2.85 (4.15)	4.12 (4.30)	*F* = 2.52, *p* = 0.086	(Comp = Unrel) > Loc
AoA	6.19 (1.82)	6.00 (1.83)	4.77 (1.60)	*F* = 8.23, *p* = 0.001	(Comp = Loc) > Unrel
**PAIR VARIABLES**					
FAS	0.053 (0.13)	0.054 (0.14)	0.000 (0.000)	*F* = 3.54, *p* < 0.05	(Comp = Loc) > Unrel
BAS	0.019 (0.05)	0.015 (0.05)	0.000 (0.000)	*F* = 2.87, *p* = 0.06	(Comp = Loc) > Unrel
Integrative Rating	5.48 (0.95)	5.50 (1.00)	1.77 (0.40)	*F* = 324.24, *p* < 0.001	(Comp = Loc) > Unrel
Visual Exp. Rating	3.02 (0.55)	3.23 (0.55)	–	*F* = 7.18, *p* = 0.01	Loc > Comp
bi_N_gram	10.33 (1.85)	10.84 (1.85)	5.60 (3.01)	*F* = 85.38, *p* < 0.001	(Comp = Loc) > Unrel

*ELP, English Lexicon Project; CD, Contextual Diversity; Ortho N, Orthographic Neighborhood; AoA, Age of Acquisition; FAS, Forward Association Strength; BAS, Backward Association Strength; Integrative ratings (1 to 7 scale); Visual Experience Ratings (0 to 5 scale); bi_N_gram, bi-directional N-gram, (co-occurrence measure)*.

### Procedure

**Figure 1:**
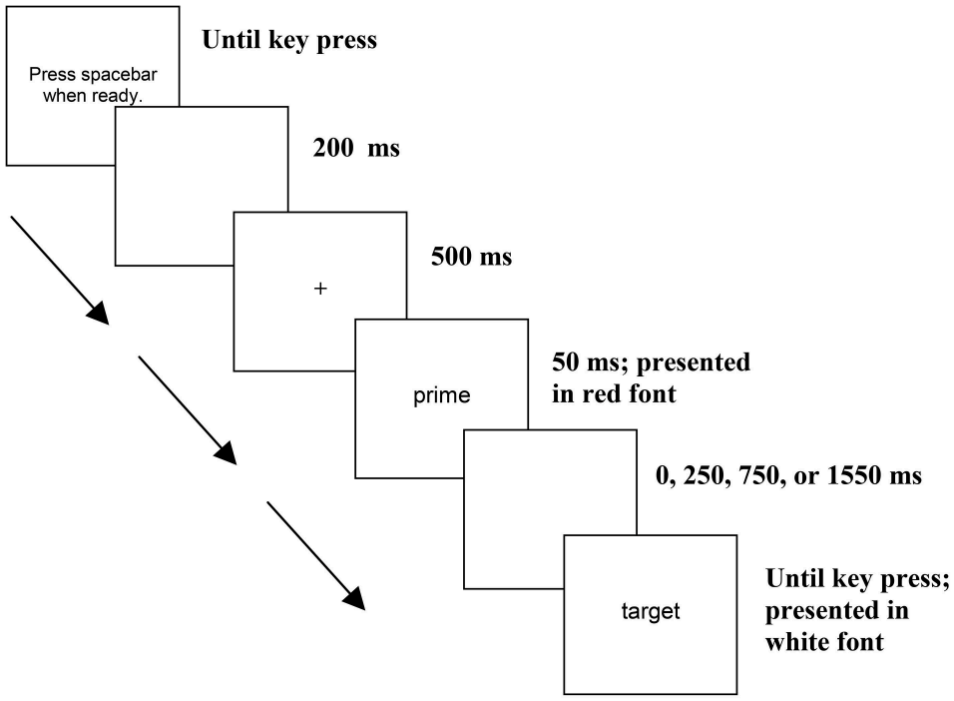
**Lexical decision task procedure**.

Trials consisted of 48 real word targets following a locative prime (16 trials), a compositional prime (16 trials) or an unrelated prime (16 trials). Across three counterbalanced experimental lists, targets were presented with each of the three prime-types. The experiment consisted of an additional 48 filler trials containing a real word prime followed by a non-word target (e.g., *cell–hife*). Primes were presented in the center of a black screen, in 22-point red Arial font, and target words were presented in white font of the same size and type. Participants responded to only the target words. Figure 1 depicts the stimuli presentation within a trial. To begin each trial, participants hit the spacebar. A blank screen appeared for 200 ms, followed by a fixation symbol (+) for 500 ms. Next the prime word appeared for 50 ms followed immediately by the target in the 50 ms SOA condition and or by a blank screen for 250, 750, or 1,550 ms in the 300, 800, and 1,600 ms SOA conditions, respectively, and then the target. Targets remained on the screen until participants indicated whether the item was a real word by pressing the J key for “yes” or the F key for “no.” For all conditions, the presentation order of the items was randomized, and there was an inter-trial interval of 1,000 ms. Participants were given 10 practice trials before completing the 96 experimental trials.

## Results and discussion

RTs from incorrect trials (1.93%) were excluded from the analyses, as well as RTs ±2.5 SDs from the mean (an additional 4.65% of the data). Prior to analyses, RTs (in milliseconds) were standardized (zRTs) by transforming each RT into a standard score based upon the participant's overall RT as done in Hutchison et al. (2008). Means and standard deviations for RTs, zRTs, and accuracies are included in Table 2. As shown in Table 1, all the prime characteristics were equivalent between the compositional and locative relations with the exception of contextual diversity and orthographic neighborhood. So we included these two prime variables in addition to the standardized baseline (ELP) target response times (zRTs) as control variables in our analyses.

**Table 2:** **Means and standard deviations of target RTs, zRTs, and accuracies**.

	**50 ms SOA**			**300 ms SOA**		
**Relation**	**RT**	**zRT**	**Acc**	**RT**	**zRT**	**Acc**
Unrelated	691 (166)	0.075 (1.03)	0.969 (0.174)	644 (160)	0.117 (1.05)	0.967 (0.179)
Compositional	672 (152)	−0.042 (0.97)	0.983 (0.130)	624 (146)	−0.064 (.91)	0.985 (0.120)
Locative	675 (154)	−0.028 (0.96)	0.978 (0.146)	623 (149)	−0.044 (1.00)	0.981 (0.135)
	**800 ms SOA**			**1,600 ms SOA**		
Unrelated	689 (204)	0.070 (0.99)	0.974 (0.159)	689 (186)	0.073 (0.99)	0.982 (0.133)
Compositional	672 (191)	−0.042 (0.94)	0.989 (0.104)	675 (174)	−0.035 (0.97)	0.992 (0.088)
Locative	676 (203)	−0.020 (1.03)	0.982 (0.132)	674 (173)	−0.033 (0.99)	0.988 (0.108)

Standardized target response times (zRTs) were analyzed using a mixed-effects regression analysis with crossed random effects for participants and items (Baayen et al., 2008). Version 3.0.2 of the R statistical language (R core team, 2013) and version 2.0-6 of the *lmerTest* package (Kuznetsova et al., 2014) were used. The *lmerTest* packages uses the Satherthwaite approximation for degrees of freedom, which allows for significance testing.

Step 1 of the analysis contained prime-type and SOA, as well as each target's standardized RT from the English Lexicon Project, each prime word's standardized orthographic neighborhood size, and each prime word's log-transformed contextual diversity. Data were coded so that the 300 ms SOA was the reference level (i.e., all other levels were statistically compared to that SOA). For prime-type, “Unrelated” was the reference level. Table 3 shows the individual standardized regression coefficients and 95% confidence intervals for this analysis, as well as the analogous accuracy analysis. Targets with slower RTs in the ELP dataset had slower RTs here as well. There was also a significant effect of prime-type, *F*_(2, 15786)_ = 29.88, *p* < 0.001. Both related prime-types produced significantly faster RTs than the Unrelated condition.

**Table 3:** **Summary of mixed-effects analysis for reaction time and accuracy**.

**Variable**	**Reaction time**		**Accuracy**	
	**β**	**95% *CI***	**β**	**95% *CI***
**STEP 1**				
Target ELP zRT	0.991^***^	[0.481, 1.501]	0.494^**^	[−0.774, −0.213]
Prime orthographic N	−0.003	[−0.022, 0.016]	0.013	[−0.006, 0.031]
Prime CD	0.002	[−0.017, 0.020]	−0.013	[−0.032, 0.005]
Prime-type: Compositional^a^	−0.127^***^	[−0.166, −0.088]	0.090^***^	[0.052, 0.129]
Prime-type: Locative^a^	−0.124^***^	[−0.161, −0.087]	0.072^***^	[0.035, 0.109]
SOA: 50^b^	−0.000	[−0.042, 0.041]	−0.009	[−0.061, 0.043]
SOA: 800^b^	−0.002	[−0.045, 0.041]	0.031	[−0.022, 0.085]
SOA: 1600^b^	−0.003	[−0.048, 0.041]	0.071^*^	[0.015, 0.126]
**STEP 2^C^**				
Compositional @ SOA 50	0.054	[−0.048, 0.155]	−0.026	[−0.128, 0.075]
Locative @ SOA 50	0.048	[−0.054, 0.150]	−0.031	[−0.133, 0.072]
Compositional @ SOA 800	0.044	[−0.060, 0.149]	−0.016	[−0.121, 0.089]
Locative @ SOA 800	0.067	[−0.039, 0.172]	−0.038	[−0.144, 0.067]
Compositional @ SOA 1600	0.075	[−0.033, 0.183]	−0.057	[−0.166, 0.052]
Locative @ SOA 1600	0.058	[−0.051, 0.166]	−0.059	[−0.168, 0.050]

* *p < 0.05*,

** *p < 0.01*,

*** *p < 0.001*.

a *Coefficient is relative to the reference level (Unrelated)*.

b *Coefficient is relative to the reference level (SOA = 300)*.

c *Coefficients are relative to the combination reference level (Unrelated, SOA = 300)*.

Step 2 of the analysis contained the prime-type × SOA interaction, which was not significant, *F*_(6, 15742)_ = 0.48, *p* = 0.82. As the table shows, results of the accuracy analysis were quite consistent with the RT analysis. Targets with slower RTs in the ELP database had lower accuracies in the current study. There was again a significant effect of prime-type, *F*_(2, 16500)_ = 12.56, *p* < 0.001. Both related prime-types produced significantly higher accuracies than the Unrelated condition. The accuracy data also revealed a significant effect of SOA [*F*_(3, 350)_ = 3.54, *p* < 0.05], with accuracy in the 1,600 condition surpassing that in the reference 300 condition. As in the RT analysis, there was no hint of a prime-type × SOA interaction, *F*_(6, 16512)_ = 0.28, *p* = 0.95.

### Correlational analyses

For the compositional and locative trials, our three pair variables of interest were reliably inter-correlated (co-occurrence and visual experience *r* = 0.33, *p* < 0.001; integrative ratings and visual experience: *r* = 0.48, *p* < 0.001; co-occurrence and integrative ratings: *r* = 0.40, *p* < 0.001). Given these robust inter-correlations, we first conducted zero-order correlations to assess the extent to which each of our three pair variables were independently related to the target zRTs on the compositional and locative trials across each SOA prior to conducting regression analyses^1^. As shown in Table 4, co-occurrence and integrative ratings were related to the target zRTs for the compositional and the locative trials across the four SOAs. Consistent with the ECCo theory's prediction of a linguistic shortcut based on the co-occurrence of the prime-target integrative pair (Lynott and Connell, 2010), we found that co-occurrence emerged early as correlate of target zRTs at the 50 ms SOA. Co-occurrence remained a reliable predictor for both relations at each SOA though to a weaker extent by the 1,600 ms SOA. Consistent with the Complementary Role Activation theory and prior results (Mather et al., 2014), integrative ratings were reliably related to faster target zRTs for both relations at each SOA. Yet visual experience ratings were related to target zRTs for only the compositional pairs and only at the 300 ms SOA.

**Table 4:** **Zero-order correlations with standardized target RTs**.

		**SOA**			
		**50 ms**	**300 ms**	**800 ms**	**1,600 ms**
**Compositional Items**	***df***	**1585**	**1156**	**1385**	**1213**
Bidirectional N-gram (co-occurrence)		−0.13^***^	−0.22^***^	−0.10^***^	−0.11^***^
Integrative Ratings		−0.08^**^	−0.12^***^	−0.08^**^	−0.07^*^
Visual Experience Ratings		−0.01	−0.15^***^	−0.03	−0.03
**Locative Items**	***df***	**1583**	**1159**	**1377**	**1213**
Bidirectional N-gram (co-occurrence)		−0.17^***^	−0.14^***^	−0.14^***^	−0.08^**^
Integrative Ratings		−0.10^***^	−0.10^***^	–0.10^***^	−0.06^*^
Visual Experience Ratings		−0.03	−0.04	−0.04	−0.02

* p ≤ 0.05;

** *p ≤ 0.01*,

*** *p ≤ 0.001*.

### Regression analyses

As in Jones and Golonka (2012), we conducted hierarchical regression analyses in order to determine whether our pair variables of interest predicted target zRTs above and beyond prime and target item control variables. Our three control variables (prime contextual diversity, prime orthographic neighborhood, and baseline ELP target zRTs) were entered into the first block of our regression model with our three predictors (co-occurrence, integrative ratings, visual experience ratings) entered into the second block. Table 5 shows the total proportion of variance accounted for in the second model, the change in *R*^2^, and the standardized betas for the compositional vs. locative trials at each SOA. Within each relation and across all SOAs, the baseline (ELP) target zRTs unsurprisingly accounted for a significant portion of the variation in our primed target zRTs. Notably, however, our three pair variables collectively accounted for a significant portion of this variation in target zRTs within each relation and across the 50, 300, and 800 ms (but not the 1,600 ms) SOAs, as exhibited by the reliable *R*^2^ changes. For both relations at the 50 ms SOA, integrative ratings remained as the only additional reliable predictor, though with a non-significant trend toward co-occurrence as a predictor. This further affirms the rapid occurrence of relational integration and provides support that relational integration facilitates lexical decisions of targets-at least for these compositional and locative integrative relations. Across both relations, integrative ratings re-emerged as a reliable predictor of faster target zRTs at the 800 ms SOA. Co-occurrence fully emerged as a reliable predictor of faster target zRTs at the 300 ms SOA for both relations and remained a reliable predictor at the 800 ms SOA for the locative but not the compositional relations. In addition to these commonalities, there was also a notable difference between the compositional and locative pairs. Visual experience ratings predicted faster target zRTs only for the compositional pairs and only at the 300 ms SOA. In contrast, for the locative pairs at this 300 ms SOA, there was not even a hint of visual experience ratings as a predictor. Thus, both the correlation and regression results suggest that visual simulation of the prime-target pair facilitates the processing of an object's material but does not facilitate processing an object within its locative context.

**Table 5:** **Regression analyses**.

**Relation**	**SOA**	**Overall model (Block 2)**	***R*^2^ Change from Block 1**	**Predictor**	**Beta**	***t***	***p***
Compositional	50	*R*^2^ = 0.040, *F*_(6, 1578)_ = 11.06, *p* < 0.001	Δ*R*^2^ = 0.009, *F* = 5.19, *p* = 0.001	Prime CD	−0.034	−1.22	0.223
				Prime Ortho-N	−0.038	−1.32	0.186
				ELP Target zRT	0.148	5.20	<0.001
				Co-occurrence	−0.055	−1.47	0.141
				Integ. Rating	−0.069	−2.08	<0.05
				Visual Exp. Rating	0.024	0.77	0.441
	300	*R*^2^ = 0.066, *F*_(6, 1149)_ = 13.51, *p* < 0.001	Δ*R*^2^ = 0.039, *F* = 15.98, *p* < 0.001	Prime CD	0.013	0.43	0.670
				Prime Ortho-N	−0.053	−1.60	0.109
				ELP Target zRT	.126	3.80	<0.001
				Co-occurrence	−0.102	−2.37	<0.05
				Integ. Rating	−0.039	−1.00	0.316
				Visual Exp. Rating	−0.104	−2.89	<0.01
	800	*R*^2^ = 0.032, *F*_(6, 1378)_ = 7.68, *p* < 0.001	Δ*R*^2^ = 0.008, *F* = 3.62, *p* = 0.01	Prime CD	0.037	1.27	0.200
				Prime Ortho-N	−0.013	−0.43	0.660
				ELP Target zRT	0.151	4.91	<0.001
				Co-occurrence	0.007	0.19	0.850
				Integ. Rating	−0.083	−2.38	<0.05
				Visual Exp. Rating	−0.026	−0.78	0.436
	1600	*R*^2^ = 0.035, *F*_(6, 1206)_ = 6.73, *p* < 0.001	Δ*R*^2^ = 0.005, *F* = 2.16, *p* = 0.09	Prime CD	−0.003	−0.09	0.928
				Prime Ortho-N	−0.012	−0.38	0.708
				ELP Target zRT	0.169	5.16	<0.001
				Co-occurrence	−0.011	−0.27	0.788
				Integ. Rating	−0.058	−1.53	0.127
				Visual Exp. Rating	−0.018	−0.52	0.605
Locative	50	*R*^2^ = 0.064, *F*_(6, 1539)_ = 17.49, *p* < 0.001	Δ*R*^2^ = 0.013, *F* = 7.18, *p* < 0.001	Prime CD	−0.023	−0.86	0.391
				Prime Ortho-N	0.064	2.34	<0.05
				ELP Target zRT	0.157	5.20	<0.001
				Co-occurrence	−0.090	−3.10	<0.01
				Integ. Rating	−0.085	−2.64	<0.01
				Visual Exp. Rating	0.031	0.90	0.367
	300	*R*^2^ = 0.050, *F*_(6, 1124)_ = 9.87, *p* < 0.001	Δ*R*^2^ = 0.009, *F* = 3.75, *p* = 0.01	Prime CD	−0.046	−1.42	0.155
				Prime Ortho-N	0.090	2.80	<0.01
				ELP Target zRT	0.114	3.22	<0.001
				Co-occurrence	−0.077	−2.26	<0.05
				Integ. Rating	−0.070	−1.82	0.069
				Visual Exp. Rating	0.026	0.65	0.518
	800	*R*^2^ = 0.032, *F*_(6, 1343)_ = 7.29, *p* < 0.001	Δ*R*^2^ = 0.018, *F* = 8.18, *p* < 0.001	Prime CD	0.058	1.96	0.050
				Prime Ortho-N	0.024	0.82	0.410
				ELP Target zRT	0.074	2.27	<0.05
				Co-occurrence	−0.107	−3.40	0.001

## General discussion

Our study is the first to compare and contrast the priming magnitude, time course, and underlying predictors of interest (co-occurrence, integrative ratings, and visual perceptual experience) for the two specific and ubiquitous compositional and locative integrative relations. Thus, our direct comparison of these relations rather than using a broad mixture of integrative relations addresses the importance of focusing on specific rather than broad relational categories (McRae and Boisvert, 1998). Although, we found no differences in the magnitude and time course of compositional vs. locative priming, as described further, our results have several implications for both the ECCo and Complementary Role Activation theories and suggest several future directions for both behavioral and neuroscientific research on these relations.

### Theoretical implications

The early emergence of co-occurrence as a correlate and predictor at the 50 ms SOA supports the linguistic shortcut posited by the ECCo theory. It is also consistent with findings of such a linguistic shortcut in a sensibility judgment task for novel conceptual combinations (e.g., *octopus apartment*; Connell and Lynott, 2013). Notably, for the compositional pairs, co-occurrence was a reliable correlate and trended toward being a reliable predictor of faster target zRTs at 50 ms, prior to the emergence of visual simulation as a correlate and predictor at 300 ms. Thus, our findings support the ECCo theory's claim and prior findings of not only a general linguistic shortcut, but one in which linguistic information (co-occurrence) begins to emerge *prior to* simulating the prime-target pair (Louwerse and Jeuniaux, 2010; Lynott and Connell, 2010; Louwerse and Connell, 2011; Louwerse and Hutchinson, 2012). This earlier emergence is likely attributable to the faster retrieval process for linguistic information in comparison to the situated simulation of the object and that object's material (e.g., a *velvet curtain* as opposed to a *plastic curtain* or *steel curtain*, etc.). Based on our regression results across the four SOAs, both co-occurrence and visual simulation peaked at 300 ms before declining by 800 ms. However, it is entirely possible that the activation peak for co-occurrence may have occurred earlier (between 50 and 300 ms) than for visual simulation as posited by the LASS theory (Barsalou et al., 2008). Additional SOAs between 50 and 300 ms and between 300 and 800 ms are needed to better pinpoint the initial emergence, peak, and decline of activation for the linguistic (co-occurrence) *vs*. the visual simulation processes.

Recall that regardless of the task (linguistic vs. spatial), both linguistic and perceptual simulation processes may be involved (Louwerse and Jeuniaux, 2010). Our results further support this by demonstrating an influence of visual simulation within the highly linguistic task of lexical priming. Yet, the limitation of this influence to only our compositional pairs suggests that the type of conceptual relation also influences the extent of linguistic vs. perceptual simulation. The finding that perceptual experience was a correlate and predictor for only the compositional pairs may reflect a more narrow focus on the object itself rather than the object within its context (e.g., the materials composing a house rather than the location of it).

For both relations, co-occurrence and integrative ratings similarly predicted faster target zRTs across the four SOAs above and beyond our control variables at the 50 and 300 ms SOAs for both relations (and at the 800 ms SOA for the locative pairs). Integrative ratings remained a reliable correlate of the zRTs for both relations through the 800 ms SOA, thereby further supporting relational integration as one mechanism of lexical priming. Co-occurrence did not precede the onset of relational integration as it did perceptual simulation. Rather both co-occurrence and relational integration processes each predicted faster target zRTs at the early 50 ms SOA. Thus, our results demonstrate an even earlier emergence of integrative priming for both the compositional and locative relations (50 ms SOA as opposed to our previously used short 100 ms SOA; Estes and Jones, 2009; Jones and Golonka, 2012). The relatively later re-emergence of integrative ratings as a predictor of faster target zRTs for both relations at the 800 ms SOA suggests at least a partially prospective nature of complementary role activation. With an 800 ms SOA, participants would have sufficient time (>300 ms; Hutchison et al., 2001; Jones, 2012) to prospectively anticipate target objects (*table*) that could be located on the prime (*patio*). However, to fully evaluate the prospective nature of integrative priming for locative and compositional (and other) integrative relations, future studies should incorporate paradigms that are more prospective in nature than the LDT, such as a continuous LDT (Jones, 2010), the speeded word fragment task (Heyman et al., 2015) or examination of the N400 effect, with larger amplitudes indicating expectancy violations in event-related brain potential (ERP). For example, larger N400 effects were found when participants read sentences containing low expectancy (but still plausible) locations (e.g., pond) in comparison to high expectancy locations (e.g., ocean) following a verb (e.g., snorkeling) that suggested an ongoing event (Ferretti et al., 2007). Similarly, for a given location prime (*patio*) there may be larger N400 effects for less integrative (but still plausible) targets (*toy*) in comparison to more easily anticipated target objects (*table*).

### Implications for neural representation of compositional and locative relations

Our current results, using only behavioral measures, provides initial support for a difference in the processing of compositional vs. locative relations (i.e., visual experience as a predictor for compositional but not locative relations). Future studies using neuroimaging methods could further differentiate between these two ubiquitous relations. Relational knowledge is thought to be comprehended via a distributed network, which consists of the dorsolateral prefrontal, posterior parietal, and the lateral temporal occipital cortices. Damage to these regions is associated with comprehension deficits (Tranel et al., 2003). Prior studies investigating the neural basis of relational knowledge have focused on the neural origins of different relation types, including locative (Wu et al., 2007), and compositional (Moss et al., 2005; Bright et al., 2007). For compositional relations, an anterior portion of the medial temporal cortex is involved in processing “fine-grained” visual information about particular objects (Bright et al., 2007), such as identifying typical properties of that object (e.g., recognizing that a tree has branches; Tyler et al., 2004). Damage to the anterior medial temporal cortex aids in processing the intrinsic features of an object, which are vital for instantiating compositional relations (e.g., stocking–wool; Muehlhaus et al., 2014). Differing degrees of perceptual simulations may entail different levels of anterior medial temporal cortical involvement in processing the visual properties of these objects. So then, this region may support the integrative priming of compositional pairs. For locative relations, the temporo-parietal junction (TPJ), inferior frontal gyrus (IFG) and supramarginal gyrus (SMG) have been implicated as vital regions for comprehension. Damage to these regions is associated with deficits in comprehending locative relations (Tranel and Kemmerer, 2004; Wu et al., 2007). Wu et al. (2007) found an association between damage to the TPJ and a deficit for locative relations. Accordingly, we would anticipate a greater involvement of the TPJ in the processing of locative compared to compositional relations.

### Limitations and additional future directions

Our relatively shallow LDT task likely amplified the extent to which co-occurrence related to/predicted our target latencies. Indeed, prior studies (Louwerse and Jeuniaux, 2010; Connell and Lynott, 2013) demonstrated an effect of task type with a bias toward linguistic processing in more verbal and shallow tasks and a bias toward perceptual simulation for tasks requiring deeper conceptual processing. Thus, future studies could use a variety of paradigms to further assess the relative contributions of the linguistic shortcut (co-occurrence), relational integration, and perceptual simulation in the processing of integrative prime-target pairs. Another limitation of our study was the inclusion of the unrelated pairs in our integrative ratings. As noted by Jouravlev and McRae (2016), who used production norms to assess thematic relatedness, item ratings are influenced by the other items on the list. So then the inclusion of unrelated items could have artificially inflated and compressed the integrative ratings of our compositional and locative pairs. The development of additional measures of integration, such as production norms may better capture the variation in integratability. For the current study, we developed and used a set of only 48 targets each having a compositional, locative, and unrelated prime. For use in future priming, memory, and neuroscience studies investigating compositional and locative relations, we are in the final stages of developing a larger stimulus set consisting of 100 + targets.

Despite these limitations, our current study represents an important first step in comparing and contrasting two highly ubiquitous types of integrative relations. Moreover, our study bridged prior research on perceptual simulation in conceptual combination with the Complementary Role Activation processes posited for integrative priming. Future studies directly comparing compositional and locative relations not only will serve to further inform embodied and integrative theories, but also will further functional magnetic resonance imaging (fMRI) research focusing on item, context, and relational encoding (Davachi, 2006). Presumably, compositional pairs would likely be supported by cortical structures involved in processing the material composing given objects, whereas locative pairs would be supported by different cortical structures responsible for encoding context.

## Ethics statement

Institutional Review Board, Division of Research, Wayne State University. We were awarded expedited approval on September 11, 2012 (IRB#087412B3E). Participants volunteered for this study to earn partial course credit toward their psychology courses by signing up for an appointment time via our online SONA participant pool. Prior to the experiment, participants read a research information sheet describing the study. As explicitly stated on this sheet, participants had the option to decline participation at any point during the study.

## Author contributions

LJ contributed to the conception, design, data analysis, data interpretation, and drafting of this work. LW contributed to the data analyses and drafting of the manuscript. RC contributed to the data acquisition, data analysis, and drafting of the manuscript. NO contributed to the data interpretation and drafting of the manuscript.

### Conflict of interest statement

The authors declare that the research was conducted in the absence of any commercial or financial relationships that could be construed as a potential conflict of interest.

## Footnotes

^1^Similar results were obtained for correlations on the item means though the small set of items (48 per relation) did not provide sufficient power to reach conventional levels of statistical significance.

## Appendix

### Stimuli

**Table:**

**Target**	**Prime**		
	**Compositional**	**locative**	**Unrelated**
ball	rubber	backyard	carrot
bed	foam	hotel	soup
bell	gold	church	blanket
bench	pine	park	milk
box	cardboard	closet	moon
bracelet	string	ankle	book
bread	pumpkin	bakery	nail
bridge	rope	city	glove
building	brick	campus	diamond
bunny	chocolate	garden	roof
cabin	concrete	mountain	silk
cage	aluminum	zoo	cloud
cake	strawberry	party	computer
candy	caramel	counter	ceiling
car	plastic	garage	cotton
chair	wicker	lawn	hand
cider	peach	mill	hair
coat	wool	lab	spoon
coin	silver	pocket	nylon
curtain	velvet	theater	hammer
desk	oak	library	puppy
fence	chain	school	cheese
floor	tile	office	pear
fort	snow	beach	button
fountain	rock	lobby	cracker
house	log	island	necklace
locker	metal	gym	dream
nest	leaf	tree	cookie
pencil	lead	drawer	coffee
pillow	feather	sofa	fruit
plane	paper	airport	grape
rack	copper	oven	heart
road	gravel	country	cabinet
saddle	leather	stable	radio
sandwich	meat	deli	door
sculpture	ice	gallery	collar
sidewalk	asphalt	neighborhood	magazine
sign	neon	street	butter
sink	granite	bathroom	phone
statue	marble	museum	magic
stool	wood	bar	machine
stove	iron	kitchen	apple
swing	tire	playground	donkey
table	stone	patio	nose
tractor	steel	farm	dog
vase	ceramic	shelf	goat
window	glass	bedroom	towel
